# Daflon Enhances Morphine Analgesia and Mitigates Tolerance in a Rat Neuropathic Pain Model

**DOI:** 10.3390/ph18101513

**Published:** 2025-10-09

**Authors:** Lokesh Kumar Mende, Meng-Lin Lee, Yaswanth Kuthati, Shu-Yi Koh, Chih-Shung Wong

**Affiliations:** 1 Department of Anesthesiology, Cathay General Hospital, Taipei 106438, Taiwan; lokeshyv66@gmail.com (L.K.M.); yaswanthk1987@gmail.com (Y.K.); kohshuyii@gmail.com (S.-Y.K.); 2 Division of Cardiovascular Surgery, Department of Surgery, Cathay General Hospital, Taipei 106438, Taiwan; martin25_lee@hotmail.com; 3 School of Medicine, National Tsing Hua University, Hsinchu 30013, Taiwan; 4 Graduate Institute of Medical Sciences, National Defense Medical University, Taipei 11490, Taiwan

**Keywords:** Daflon, intrathecal, morphine, tail flick test, tolerance, anti-nociceptive effect, pain management, pain threshold, anti-inflammatory, antioxidant

## Abstract

**Objective:** Morphine is a widely used analgesic for severe pain, but tolerance is a major challenge in long-term pain management. This study examined the potential of Daflon^®^ to enhance morphine’s pain-relieving effects and to reduce tolerance in a rat model with neuropathic pain induced by partial sciatic nerve transection (PSNT). **Methods:** Male Wistar rats were divided into five groups: (1) Sham + Saline, (2) PSNT + Saline, (3) PSNT + morphine, (4) PSNT + Daflon, and (5) PSNT + morphine + Daflon. Morphine tolerance was induced through continuous intrathecal infusion (15 µg/µL/h, i.t.) for 7 days, starting on day 7 post-PSNT, while Daflon was administered orally (50 mg/kg/day, oral) for 7 days. Pain relief was assessed using tail-flick and paw withdrawal on days 1, 4, and 7 after osmotic pump implantation. Spinal cords were collected for immunohistochemistry to analyze glial expression, and serum biomarkers (TNF-α, IL-1β, IL-6, and IL-10) were measured to evaluate neuroinflammation. **Results:** The results showed that oral Daflon significantly enhanced morphine’s analgesic effects, evidenced by improved pain thresholds in all behavioral tests. Moreover, Daflon reduced morphine tolerance. Mechanistically, Daflon upregulated the expression of nuclear factor erythroid 2-related factor 2 (Nrf2) and activated heme oxygenase-1 (HO-1), reducing oxidative stress and modulating neuroinflammation through glial regulation. Combining morphine and Daflon reduces pro-inflammatory cytokines (TNF-α, IL-1β, and IL-6) and enhances anti-inflammatory IL-10 serum level, showing a synergistic effect in managing neuropathic pain with greater efficacy and lower drug dependence. Histology and immunohistochemistry evaluations further confirmed that morphine and Daflon co-treatment substantially reduced mononuclear cell infiltration, astrocyte activation (as indicated by GFAP expression), and microglial activation (as indicated by Iba-1 expression) compared to single treatment. **Conclusions:** Our findings suggest that dual therapy synergistically targets both oxidative stress and inflammatory pathways, leading to stronger neuroprotection and pain relief. Importantly, the combination approach may allow for lower opioid dosages, minimizing the risks of opioid-related side effects. Overall, morphine and Daflon co-administration offers a promising and safer strategy for managing neuropathic pain and preserving spinal cord integrity.

## 1. Introduction

Diosmin is used in clinical management of chronic venous insufficiency and varicose veins due to its phlebotonic and vascular-protective properties, primarily through the inhibition of noradrenaline uptake and metabolism in venous walls [1]. Flavonoids, a class of natural compounds, are well known for their beneficial anti-hyperalgesic effects [2]. Daflon, also known as micronized purified flavonoid fraction (MPFF), is an oral phlebotonic flavonoid combination containing diosmin and hesperidin (9:1) that is commonly used clinically for the management of venous disorders [3]. In this study, we refer to MPFF using its commercial name, Daflon. After oral administration, diosmin is converted to diosmetin, which is subsequently absorbed and esterified into glucuronide conjugates that are excreted in the urine. The pharmacological effects of diosmin have been investigated in several in vitro and in vivo studies, and it was found to possess anti-inflammatory, antioxidant, antidiabetic, antihyperlipidemic, and antifibrotic effects in different disease models and clinical studies [4,5]. Toxicological studies showed that diosmin has a favorable safety profile. Accordingly, diosmin is a potentially effective and safe treatment for many diseases [4]. In vitro and in vivo research have shown that diosmin has antihyperglycemic, anti-inflammatory, antioxidant, antimutagenic, and anti-ulcer properties. Diosmin is beneficial for inflammatory conditions it lowers the levels of IL-1, IL-8, and NO while increasing the level of IL-10 [6]. The therapeutic potential of diosmin was also explored in neuropathic pain models. Many biological roles of diosmin may contribute to its therapeutic significance. Daflon, containing diosmin, has been used to treat vascular illnesses such as chronic venous diseases (CVD) in clinical settings [1,3,7]; however, its therapeutic potential in clinical pain management has not been explored.

Inflammation is an intrinsic immune response against pathogens, damaged cells, and other potentially harmful substances. Although inflammation is necessary for the immune system, excessive inflammatory reactions can lead to serious disorders such as cancer, chronic asthma, and arthritis. Diosmin has been shown to reduce inflammatory responses in RAW264.7 macrophages activated by lipopolysaccharide (LPS) by downregulating the activity of the NF-κB and MAPK signaling pathways [8,9]. Additionally, by reducing the activity of NF-κB signaling and the levels of pro-inflammatory cytokines, such as IL-1β, TNF-α, IL-6, myeloperoxidase activity, trypsinogen activation peptide, and iNOS, diosmetin improves cerulein-induced acute pancreatitis in mice [10]. The flavonoid family have an inhibitory effect on the production of IL-1β, TNF-α, IL-6, and IL-8 in LPS-stimulated whole blood. Quercetin, naringenin, naringin, and diosmetin have this immunosuppressor effect. However, rutin and diosmin lack this anti-inflammatory regulatory effect [11].

Diosmin’s antioxidant activity plays an important role in the relief of CVD-related symptoms because oxygen-free radicals, which were generated during inflammatory processes, cause tissue damage and release of inflammatory mediators. Diosmetin also exhibits antioxidant activity in a cupric chloride (CuCl_2_)-induced plasma oxidation assay by inhibiting reactive oxygen species (ROS) generation, and attenuating malondialdehyde (MDA) formation [12]. In addition, diosmetin was demonstrated to inhibit H_2_O_2_-induced oxidative damage in L02 cells via activating the NRF2/NQO1-HO-1 signaling pathway [13]. Diosmin exerted its radio-protective effects by attenuating oxidative stress, decreasing radiation-induced DNA damage and apoptosis, boosting the antioxidant state and an-ti-peroxidative potential, therefore may have a favorable impact on radiation-induced tissue damage [14]. Diosmin has also been shown to reduce H_2_O_2_-induced chondrocyte impairment by downregulating iNOS, COX-2, IL-1β, COL1A1, MMP-3, and MMP-9, and upregulating TIMP-1, SOX9, and COL2A1 [15].

Prolonged morphine exposure activates microglial cells via various immunological signaling pathways [16]. Given the importance of spinal microglial cell activation in morphine tolerance, it is reasonable to expect that inhibiting glial cell activation will reduce morphine tolerance; this may disrupt the signaling mechanisms that cause morphine tolerance. Several drugs, including metformin, minocycline, resveratrol, and curcumin, have been demonstrated to have microglial inhibitory effect, and helpful in reducing morphine tolerance [17,18,19,20]. Morphine’s therapeutic use is restricted due to its analgesic tolerance, hyperalgesia, dependency, addiction, respiratory depression, and constipation with prolonged administration. Finding novel analgesics with fewer adverse effects is therefore essential in order to potentially boost morphine’s effectiveness while lowering its negative effects. Opioids have been utilized to provide analgesia in clinical settings for decades. Tolerance develops after long-term opioid administration. Koob and Bloom [21] proposed two pharmacological tolerance mechanisms within-system and between-system adaptation. Repeated morphine use may induce neuroadaptive alterations in the brain, resulting in drug-seeking behavior after discontinuation [22,23]. Furthermore, the nucleus accumbens and ventral tegmental area are among the primary brain regions that undergo neuroadaptive changes following morphine misuse [24]. Therefore, an opioid-sparing strategy is currently considered a mainstay in clinical pain management.

Neuropathic pain is a complex and challenging condition characterized by heightened sensitivity to pain, inflammation, and oxidative stress, often resulting from nerve injury. Current treatment options, such as opioids, provide relief but are often associated with significant side effects and long-term risks. This study explores the potential of combining morphine, a powerful analgesic, with Daflon, a flavonoid-based compound known for its anti-inflammatory and antioxidant properties, to address neuropathic pain more effectively. By targeting multiple aspects of the condition including mechanical and thermal hypersensitivity, inflammation, and oxidative stress, the morphine + Daflon aims to enhance pain relief while reducing the reliance on high-dose opioids. The findings demonstrate that this novel approach not only alleviates pain but also improves antioxidant defenses (Nrf2 and HO-1) and balances inflammatory responses, offering a promising strategy for safer and more comprehensive neuropathic pain management.

## 2. Results

### 2.1. Antinociceptive Effects of Daflon and Morphine Against Acute Pain (Tail-Flick Test)

**Figure 1:**
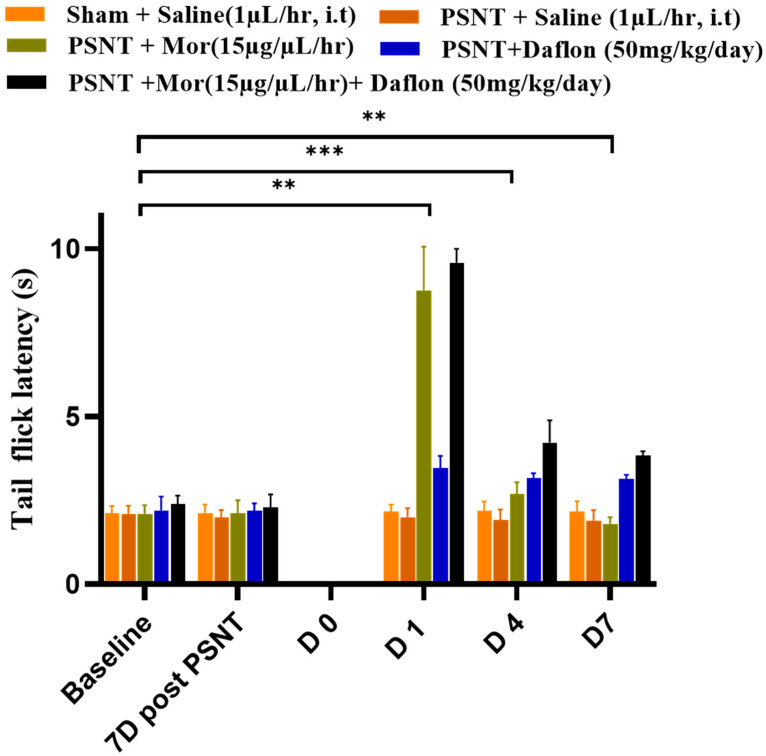
Sham + Saline (uninjured baseline), PSNT + Saline (injury control), PSNT + Mor, PSNT + Daflon, and PSNT + Mor + Daflon groups tail-flick immersion test (52 °C, hot water) on Daflon and morphine antinociception in PSNT rats. Data are presented as mean ± SD (*n* = 6). Significance: ** *p* < 0.01, *** *p* < 0.001. One-way ANOVA with Tukey’s post hoc test was used. Significance symbols: Asterisked *p* values denote Tukey-adjusted comparisons vs. PSNT + Saline; Morphine + Daflon vs. Morphine; Morphine + Daflon vs. Daflon.

Figure 1 illustrates the results of the tail-flick immersion test at 52 °C to evaluate the antinociceptive effect of morphine and daflon in PSNT rats. Morphine was intrathecally administered at a dose of (15 μg/μL/h, i.t) and Daflon (50 mg/kg/day) was orally administered for 7 consecutive days. The Sham + Saline group maintained consistent tail-flick latencies throughout, representing a stable baseline response to thermal pain in uninjured rats. In contrast, the PSNT + Saline group showed lower latencies than sham-saline control, reflecting heightened thermal sensitivity and hyperalgesia caused by nerve injury. Treatment with morphine (PSNT + morphine group) resulted in a notable increase in latency, demonstrating its effective pain-relieving properties. In contrast, Daflon treatment only showed a moderate antinociceptive effect in tail-flick latency in PSNT rats. The most significant effect was observed in the PSNT + morphine + Daflon group, where tail-flick latencies were the highest among all groups, indicating a synergistic or additive effect of the combination. These findings highlight the enhanced efficacy of combining morphine with Daflon in providing superior antinociceptive effects, which may allow for reduced morphine dosages and potentially mitigate opioid-related side effects.

### 2.2. Daflon and Morphine Alleviated PSNT Induced Thermal Hyperalgesia and Mechanical Allodynia

**Figure 2:**
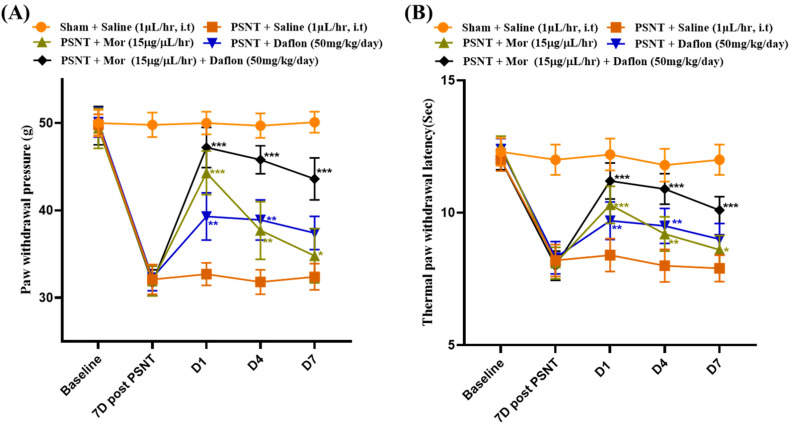
The effect of morphine and Daflon in Sham + Saline (uninjured baseline), PSNT + Saline (injury control), PSNT + Mor, PSNT + Daflon, and PSNT + Mor + Daflon. (**A**) Paw withdrawal threshold of rats in grams of weight. (**B**) Paw withdrawal to infrared radiant heat was measured to index thermal pain threshold in PSNT rats to assess the effect of continuous morphine and Daflon infusions. Data are presented as mean ± SD (*n* = 6). Significance: * *p* < 0.05, ** *p* < 0.01, *** *p* < 0.001. One-way ANOVA with Tukey’s post hoc test was used. Significance symbols: Asterisked *p* values denote Tukey-adjusted comparisons vs. PSNT + Saline; Morphine + Daflon vs. Morphine; Morphine + Daflon vs. Daflon.

Figure 2 highlights the effects of different treatments on pain sensitivity in PSNT rats. Figure 2A shows paw withdrawal thresholds to assess mechanical sensitivity. The Sham + Saline group maintained normal thresholds, while the PSNT + Saline group showed significantly reduced thresholds, indicating neuropathic pain. Morphine treatment increased thresholds, demonstrating its effectiveness in reducing pain. Daflon also improved thresholds, albeit to a lesser extent than morphine. Notably, the morphine + Daflon combination produced the highest and most sustained improvements, suggesting a synergistic effect in alleviating mechanical hypersensitivity. Figure 2B depicts withdrawal thresholds to an infra-red heat source, assessing thermal sensitivity. Similar trends were observed, with the Sham + Saline group showing normal thresholds and the PSNT + Saline group demonstrating thermal hyperalgesia. Morphine significantly improved thresholds, and Daflon provided moderate relief. The morphine + Daflon showed the most robust effects, reducing thermal sensitivity to near-normal levels. These findings highlight the enhanced efficacy of combining morphine with Daflon in managing both mechanical and thermal hypersensitivity in neuropathic pain, potentially allowing for lower morphine doses and reduced side effects.

### 2.3. Daflon and Morphine-Induced Nrf-2 and HO-1 Expression in the Dorsal Horn of PSNT Rats

**Figure 3:**
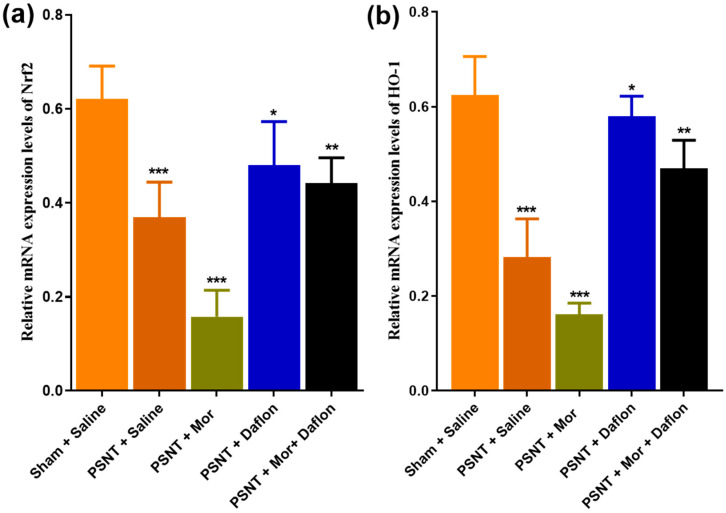
Nrf2 and HO-1 expression after PSNT and treatments. (**a**) Nrf2 and (**b**) HO-1 mRNA (qRT-PCR; relative to Sham (uninjured baseline)). PSNT reduced both markers; PSNT + Morphine showed a further decrease versus PSNT + Saline (injury control); PSNT + Daflon partially increased expression; the Morphine + Daflon combination showed the greatest restoration toward sham levels. Data are presented as mean ± SD (*n* = 6). Significance: * *p* < 0.05, ** *p* < 0.01, *** *p* < 0.001. One-way ANOVA with Tukey’s post hoc test was used. Significance symbols: Asterisked *p* values denote Tukey-adjusted comparisons vs. PSNT + Saline; Morphine + Daflon vs. Morphine; Morphine + Daflon vs. Daflon.

Figure 3 highlights the effects of different treatments on the mRNA expression levels of Nrf2 and HO-1, key components of the antioxidant defense system. Figure 3a shows that Nrf2 expression was significantly reduced in the PSNT + Saline group compared to the Sham + Saline group, indicating impaired antioxidant activity due to nerve injury. Treatment with either morphine or Daflon moderately restored Nrf2 levels, while their combination produced the most substantial increase, nearing levels seen in uninjured rats. Similarly, Figure 3b shows that HO-1 expression followed the same trend, with the combination therapy of morphine + Daflon achieving the most significant restoration, almost reaching baseline levels. PSNT reduced Nrf2 and HO-1 versus sham. Morphine alone did not restore these transcripts and showed an additional decrease relative to PSNT + Saline. Daflon alone partially increased Nrf2/HO-1, and the morphine + Daflon combination produced the greatest restoration. These results highlight the synergistic effect of combining morphine and Daflon in enhancing antioxidant defenses, offering potential to mitigate oxidative stress in neuropathic pain conditions.

### 2.4. Effect of Daflon and Morphine on Serum Cytokine Levels in PSNT Rats

**Figure 4:**
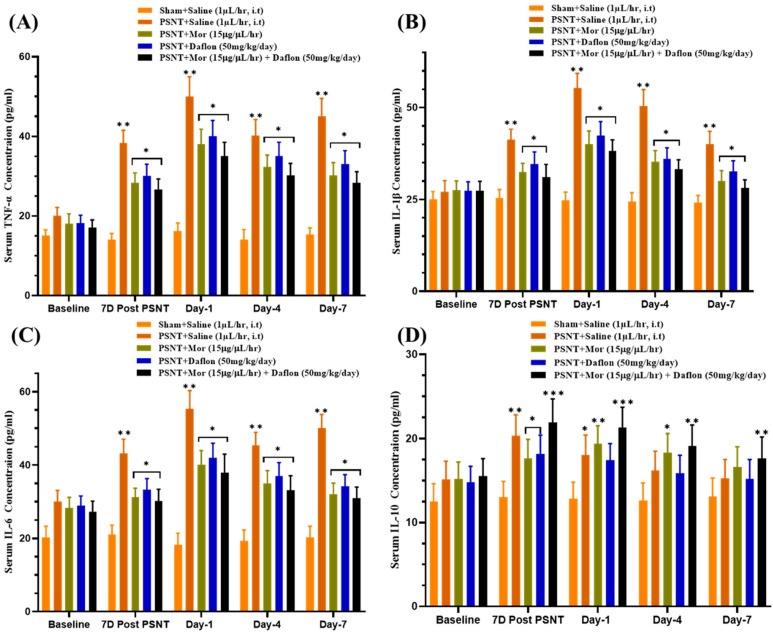
ELISA-based assessment of serum cytokines in PSNT rats across groups Sham + Saline (uninjured baseline), PSNT + Saline (injury control), PSNT + Mor, PSNT + Daflon, and PSNT + Mor + Daflon with (**A**) TNF-α (pg/mL), (**B**) IL-1β (pg/mL), (**C**) IL-6 (pg/mL), and (**D**) IL-10 (pg/mL). Data are presented as mean ± SD (*n* = 6). Significance: * *p* < 0.05, ** *p* < 0.01, *** *p* < 0.001. One-way ANOVA with Tukey’s post hoc test was used. Significance symbols: Asterisked *p* values denote Tukey-adjusted comparisons vs. PSNT + Saline; Morphine + Daflon vs. Morphine; Morphine + Daflon vs. Daflon.

Figure 4 highlights the effects of different treatments on pro- and anti-inflammatory cytokines in serum. In Figure 4A, TNF-α levels were lowest in the Sham + Saline group, while the PSNT + Saline group showed a significant increase, indicating nerve injury-induced inflammation. Both morphine and Daflon moderately reduced TNF-α levels, but their combination had the greatest effect, reducing inflammation to near-baseline levels. Figure 4B shows similar trends for IL-1β, with elevated levels in the PSNT + Saline group and moderate reductions with individual treatments. The morphine + Daflon combination demonstrated the most substantial reduction, effectively controlling systemic inflammation. Figure 4C illustrates IL-6 levels, which were also elevated in the PSNT + Saline group but decreased with treatment. The combination therapy significantly reduced IL-6 levels, approaching those seen in uninjured rats. Lastly, Figure 4D highlights IL-10, an anti-inflammatory cytokine, which was suppressed in the PSNT + Saline group. Moreover, morphine and Daflon partially restored IL-10 levels, but the morphine + Daflon significantly enhanced anti-inflammatory activity, suggesting a synergistic effect. Together, these results underscored the superior efficacy of combining morphine and Daflon in managing inflammation and promoting anti-inflammatory responses in neuropathic pain.

### 2.5. Mononucleate Cell Infiltration in the Dorsal Horn of Daflon and Morphine Induced PSNT Rats

**Figure 5:**
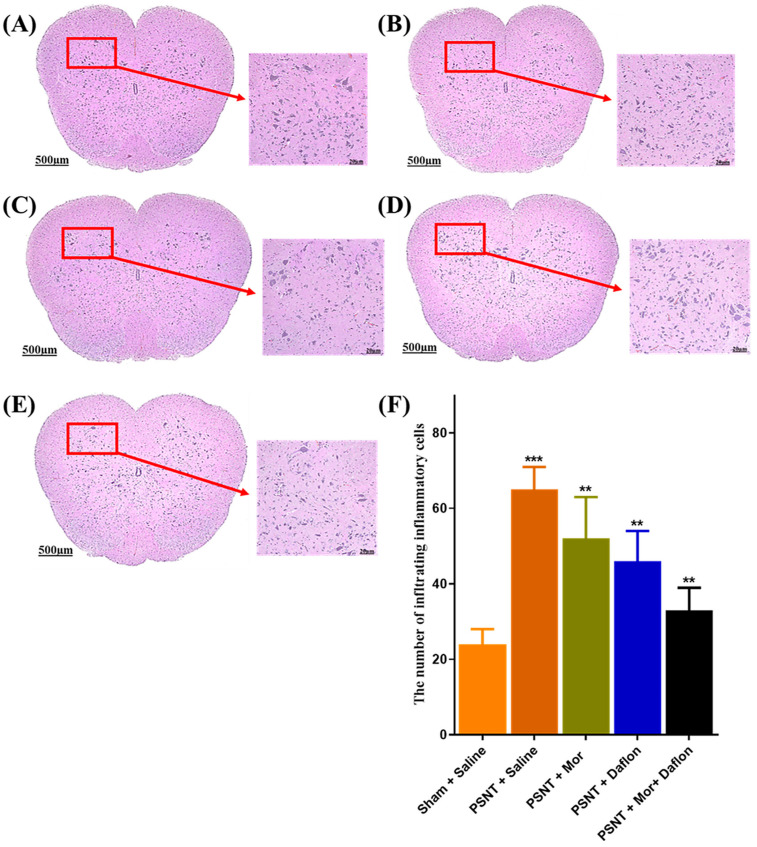
H&E staining highlights mononuclear inflammatory infiltration in (**A**) Sham + Saline (uninjured baseline), (**B**) PSNT + Saline (injury control), (**C**) PSNT + Mor, (**D**) PSNT + Daflon, (**E**) PSNT + Mor + Daflon, and (**F**) quantitative metrics. Data are presented as mean ± SD (*n* = 6). Significance: ** *p* < 0.01, *** *p* < 0.001. One-way ANOVA with Tukey’s post hoc test was used. Significance symbols: Asterisked *p* values denote Tukey-adjusted comparisons vs. PSNT + Saline; Morphine + Daflon vs. Morphine; Morphine + Daflon vs. Daflon. Scale bar = 20 µm.

Hematoxylin and eosin (H&E) staining were used to assess mononuclear cell infiltration as a marker of inflammation in the lumbar spinal cord. In the Sham + Saline group (Figure 5A), tissue structure remained intact with very little infiltration, indicating minimal inflammation. However, the PSNT + Saline group exhibited a clear increase in mononuclear cell infiltration, reflecting a strong inflammatory response after nerve injury (Figure 5B). Treatment with either morphine (Figure 5C) or Daflon (Figure 5D) alone led to a noticeable but partial reduction in infiltration compared to the PSNT + Saline group. Interestingly, the group receiving combined morphine and Daflon treatment showed greater reduction in inflammatory cell infiltration, suggesting a stronger anti-inflammatory effect (Figure 5E). These observations were supported by quantitative analysis (Figure 5F), which showed a significant rise in infiltrates in the PSNT + Saline group relative to sham controls, while all treatment groups demonstrated reduced infiltration, with the morphine + Daflon being the most effective.

### 2.6. GFAP Stained Activated Astrocytes in the Dorsal Horn of Daflon and Morphine Treated PSNT Rats

**Figure 6:**
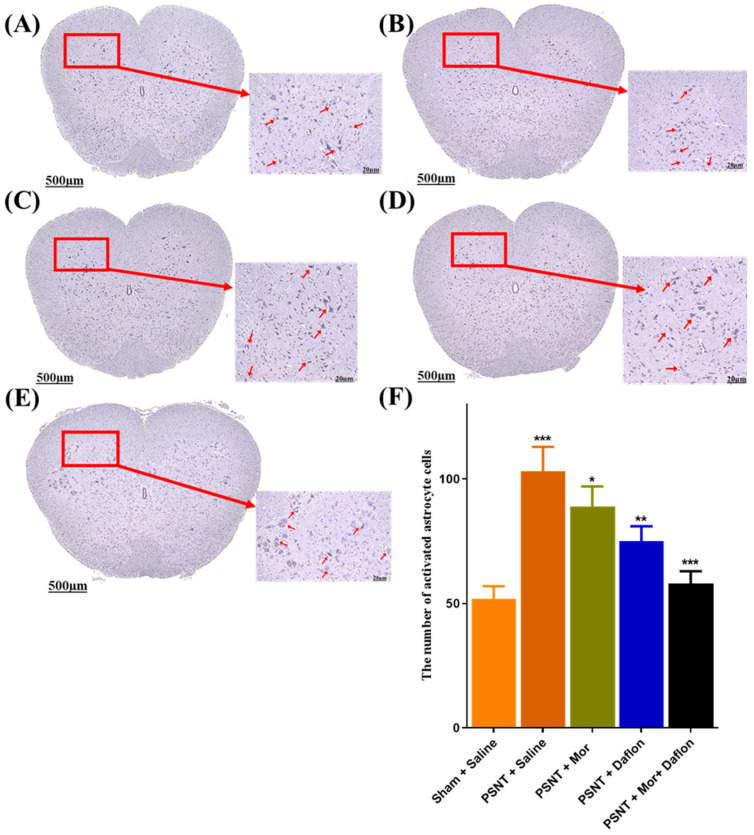
Activated astrocytes with GFAP-stained cross-sections of rat spinal cords in (**A**) Sham + Saline (uninjured baseline), (**B**) PSNT + Saline (injury control), (**C**) PSNT + Mor, (**D**) PSNT + Daflon, (**E**) PSNT + Mor + Daflon, and (**F**) quantitative analysis of activated astrocyte counts. Red arrows indicate representative GFAP-positive (activated) astrocytes in the magnified insets. Data are presented as mean ± SD (*n* = 6). Significance: * *p* < 0.05, ** *p* < 0.01, *** *p* < 0.001. One-way ANOVA with Tukey’s post hoc test was used. Significance symbols: Asterisked *p* values denote Tukey-adjusted comparisons vs. PSNT + Saline; Morphine + Daflon vs. Morphine; Morphine + Daflon vs. Daflon. Scale bar = 20 µm.

GFAP immunostaining was used to assess astrocyte activity in the dorsal horn of the lumbar spinal cord across the different experimental groups. Very few GFAP-positive astrocytes were detected, reflecting a normal resting state in the Sham + Saline group (Figure 6A). Following nerve injury, the PSNT + Saline group showed a marked increase in GFAP-positive cells, indicating strong astrocyte activation (Figure 6B). Treatment with either morphine alone (Figure 6C) or Daflon alone (Figure 6D) led to a partial decrease in astrocyte activation compared to the PSNT + Saline group. Interestingly, the combination of morphine and Daflon administration resulted in a greater reduction in GFAP-positive cells (Figure 6E). These findings were supported by quantitative analysis, which showed a significant increase in astrocyte activation in the PSNT + Saline group compared to Sham controls (*p* < 0.001). While all treatment groups reduced GFAP-positive cell expression, the group with combined therapy demonstrated the greatest reduction (Figure 6F).

### 2.7. Iba-1 Stained Activated Microglia in the Dorsal Horn of Daflon and Morphine Treated PSNT Rats

**Figure 7:**
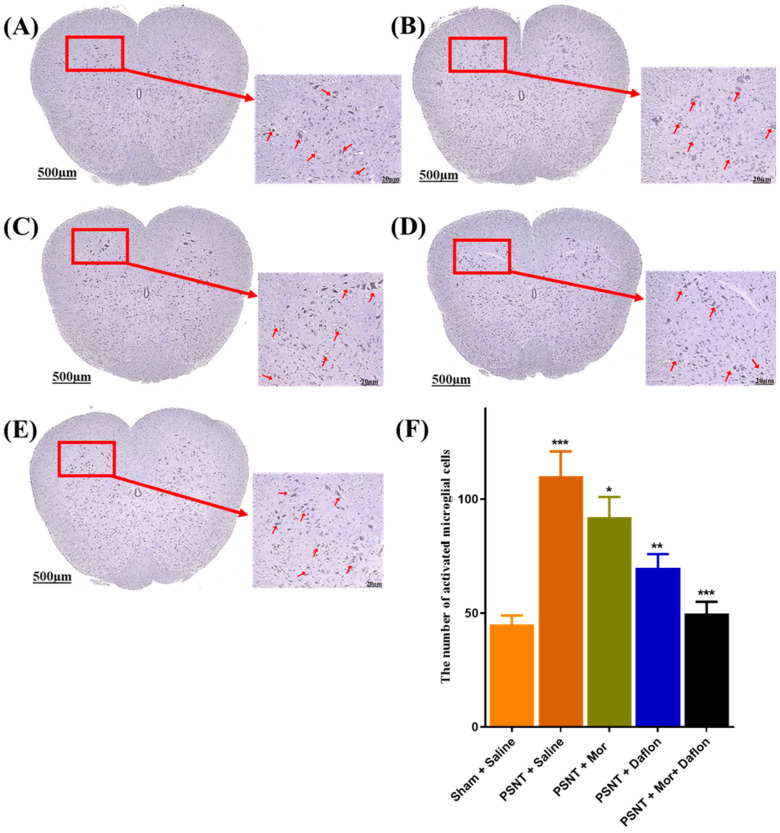
Activated microglial cells with Iba-1 antibody-stained cross-sections of rat spinal cords in (**A**) Sham + Saline (uninjured baseline), (**B**) PSNT + Saline (injury control), (**C**) PSNT + Mor, (**D**) PSNT + Daflon, (**E**) PSNT + Mor + Daflon, and (**F**) quantitative analysis of activated microglial cells. Red arrows indicate Iba-1–positive (activated) microglia in the magnified insets. Data are presented as mean ± SD (*n* = 6). Significance: * *p* < 0.05, ** *p* < 0.01, *** *p* < 0.001. One-way ANOVA with Tukey’s post hoc test was used. Significance symbols: Asterisked *p* values denote Tukey-adjusted comparisons vs. PSNT + Saline; Morphine + Daflon vs. Morphine; Morphine + Daflon vs. Daflon. Scale bar = 20 µm.

Iba-1 immunostaining was performed to evaluate microglial activation in the dorsal horn of the lumbar spinal cord across different experimental groups. In the Sham + Saline group, only a few microglial cells were observed, displaying a resting morphology (Figure 7A). Following nerve injury, the PSNT + Saline group showed a marked increase in Iba-1 positive microglia, indicating a strong activation response (Figure 7B). Treatment with either morphine (Figure 7C) or Daflon (Figure 7D) alone led to a moderate reduction in microglial activation compared to the PSNT + Saline group. Not surprisingly, the group with co-administration of morphine and Daflon exhibited a more substantial decrease in activated microglia (Figure 7E). These findings were confirmed by quantitative analysis, which showed a significant rise in microglial activation in the PSNT + Saline group compared to sham controls (*p* < 0.001). All treatment groups showed reductions, with the morphine + Daflon demonstrating the most pronounced effect (Figure 7F).

## 3. Discussions

In this partial sciatic nerve transection (PSNT) model, combining morphine with Daflon provided broader and more durable benefits than either agent alone. Across behavioral assays, the co-treatment raised tail-flick latencies and improved both mechanical and thermal thresholds, while also slowing the loss of morphine efficacy over time. At the tissue and molecular levels, these functional gains have paralleled lower glial activation in the dorsal horn (GFAP and Iba-1), reduced mononuclear infiltration, down-regulated pro-inflammatory cytokines (TNF-α, IL-1β, IL-6), and increased the anti-inflammatory cytokine IL-10. In addition, the combination also restored the antioxidant axis (Nrf2/HO-1), which is depressed after nerve injury. Taken together, the data point to a complementary interaction in which Daflon’s anti-inflammatory and antioxidant actions help preserve and even amplify morphine’s antinociception while limiting processes that drive tolerance.

Neuropathic pain engages multiple, self-reinforcing pathways: peripheral and central sensitization, neuroimmune activation, and oxidative stress. Opioids remain effective analgesics, but prolonged exposure can activate microglia and astrocytes, elevate pro-inflammatory mediators, and blunt opioid signaling, culminating in hyperalgesia and tolerance [16,17,18,19,20]. Our findings fit this framework. PSNT increased hypersensitivity and robustly activated spinal glia, whereas monotherapy with morphine or Daflon only partly reversed these changes. In contrast, the combined regimen produced the largest behavioral improvements and the greatest reductions in GFAP and Iba-1-positive cells. Clinically, this supports an opioid-sparing strategy: dampening neuroinflammation alongside opioid therapy can preserve analgesia with lower opioid exposure [25,26,27].

The cytokine profile of our present study reinforces this interpretation. Following PSNT, serum TNF-α, IL-1β, and IL-6 were elevated, consistent with systemic inflammation after nerve injury. Morphine or Daflon alone reduced these mediators modestly, but the combination shifted the balance more decisively lowering pro-inflammatory cytokines toward sham levels while increasing anti-inflammatory cytokine IL-10 expression. Because cytokines can sensitize nociceptive circuits and potentiate glial reactivity, this simultaneous decrease in pro-inflammatory signals and increase in anti-inflammatory tone is likely central to the sustained behavioral relief we observed. It also helps explain why tolerance progressed more slowly with the combination: a quieter inflammatory milieu provides fewer “failure signals” to opioid pathways.

Oxidative stress is the other pillar of this story. Nrf2 and its downstream effector HO-1 are key coordinators of endogenous antioxidant defense and cytoprotection. In our model, PSNT depressed Nrf2/HO-1 expression, while co-treatment nearly normalized both markers. Prior work has linked Nrf2 activation to reduced nociception and better opioid responsiveness [28,29]. By restoring this pathway, Daflon may stabilize redox homeostasis, protect neurons and glia from oxidative injury, and counter maladaptive plasticity that contributes to tolerance. The observed histological improvements fewer infiltrating mononuclear cells and calmer glia are consistent with this antioxidant and anti-inflammatory synergy.

It is worth emphasizing that Daflon alone showed only a moderate behavioral effect compared with morphine, yet it clearly strengthened morphine’s benefits when given together. This pattern suggests complementary rather than redundant mechanisms. Morphine primarily targets opioid receptors to blunt nociceptive transmission; Daflon appears to reshape the inflammatory and oxidative environment that normally erodes opioid efficacy. When combined, immediate antinociception is delivered by morphine, while Daflon maintains a tissue context that sustains that relief and slows tolerance. From a translational viewpoint, such division of labor is attractive: adjuncts that modulate the pain microenvironment could enable lower opioid doses without sacrificing effect size, potentially reducing adverse events associated with chronic opioid therapy.

Our histological and immunohistochemical data further support this integrative view. H&E staining showed that PSNT triggered robust dorsal-horn infiltration, a hallmark of neuroinflammation, which was attenuated most strongly by the combination. Similarly, GFAP and Iba-1 canonical indices of astrocyte and microglial activation were highest after PSNT, partly reduced by each monotherapy, and most normalized by co-treatment. These cellular changes align with the serum cytokine shifts and the restored Nrf2/HO-1 axis, forming a coherent biological narrative across behavior, blood, and spinal cord.

The results of our present study, combination of Daflon with morphine therapy, echo and extend prior reports that link glial reactivity to morphine tolerance and demonstrate that limiting microglial and astrocytic activation preserves opioid analgesia [16,17,18,19,20,25,26,27]. It also aligns with evidence that boosting antioxidant defenses through Nrf2/HO-1 activation reduces neuropathic pain behaviors and enhances opioid effects [27,29]. Notably, diosmin/diosmetin and related flavonoids have shown anti-hyperalgesic and immunomodulatory properties in preclinical models [11,30]. Our data add that, within a PSNT paradigm, pairing Daflon with morphine yields a broader, more durable benefit than either alone, consistent with a synergistic not merely additive interaction.

Limitations should be considered. We studied a single dose and schedule for each agent in male rats only; dose–response relationships, sex differences, and longer treatment windows remain to be mapped. Cytokines were measured in serum rather than cerebrospinal fluid; although serum changes tracked with spinal histology, central cytokine dynamics were not directly quantified. We assessed Nrf2/HO-1 at the transcript level; complementary protein/activity measures and pharmacologic or genetic manipulation (e.g., Nrf2 inhibition, IL-10 neutralization) would help establish causal links between these pathways and the observed behavioral protection. Finally, while PSNT is a well-established neuropathic model, replication in additional models (e.g., spared-nerve injury, chemotherapy-induced neuropathy, or diabetic neuropathy) would clarify generalizability.

With these caveats in mind, the present findings provide a practical rationale for combining Daflon with morphine for neuropathic pain management in clinical practice. By concurrently quieting neuroinflammation and restoring antioxidant capacity, Daflon appears to reinforce morphine’s antinociception and slow the processes that undermine it. In principle, such an approach could reduce the total opioid burden needed to achieve relief, improving safety without compromising efficacy. Future work should (i) explore dose-finding and timing to maximize synergy, (ii) test durability after treatment stops, (iii) evaluate central cytokines and redox markers, and (iv) probe mechanistic necessity with pathway specific inhibitors or knockdowns. If these results hold across models and conditions, adjunctive Daflon could be a low-risk, accessible tool to enhance opioid analgesia and curb tolerance in neuropathic pain. Altogether, these results highlight that a dual approach using morphine and Daflon more effectively controls neuroinflammation, limits glial activation, and helps pre-serve spinal cord integrity, offering a promising strategy for improving neuropathic pain management.

## 4. Materials and Methods

**Figure 8:**
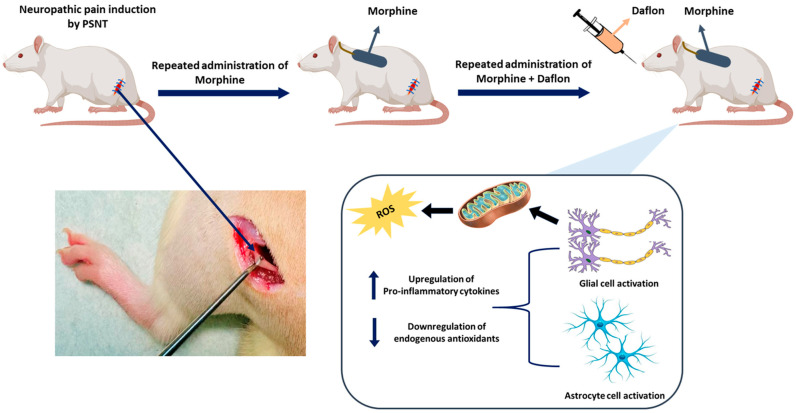
Schematic representation of the study. Morphine (15 μg/μL/h, i.t.) and Daflon (50 mg/kg/day, oral) were administered daily for 7 days.

Figure 8 describes the experimental design and mechanism of Daflon and morphine co-treatment in PSNT (Partial sciatic nerve transection)-induced neuropathic pain. PSNT induces oxidative stress and the release of pro-inflammatory cytokines, leading to the activation of glial and astrocyte cells. Morphine (15 μg/μL/h, i.t.) was administered via intrathecal infusion, and Daflon (50 mg/kg/day, oral) was given orally for 7 days. The combined treatment reduced oxidative stress, inhibited glial activation, and improved analgesic efficacy, highlighting a synergistic effect in managing neuropathic pain.

### 4.1. Establishment of the Neuropathic Pain Animal Model

This experiment used Pathogen-free male Wistar rats (350–400 g) with partial sciatic nerve transection (PSNT) as the neuropathic pain model accomplished according to previously reported procedures [31,32]. Male Wistar rats were obtained from BioLASCO, Taipei, Taiwan. In the PSNT group, the left sciatic nerve was exposed to the mid-thigh. A 7-0 polypropylene (Prolene) ligature was passed through the nerve at its midpoint, immediately cranial to the branch supplying the biceps femoris, and the ventrocranial half of the nerve was transected up to the ligature. Sham animals underwent the same exposure of the left sciatic nerve, followed by wound closure without transection. On postoperative day 7, mechanical paw-withdrawal thresholds were assessed in both sham-operated and PSNT rats. Morphine tolerance was induced by continuous intrathecal morphine infusion (15 μg/μL/h, i.t. for 7 days), and Daflon (50 mg/kg/day, oral) was co-administered orally daily to examine its effect on morphine and tolerance development. All animal procedures were approved by the Cathay General Hospital Institutional Animal Care and Use Committee (CGH-IACUC-113-010) and conducted in accordance with the hospital’s Guide for the Care and Use of Laboratory Animals.

### 4.2. Intrathecal Catheterization and Drug Administration

Daflon was administered at 50 mg/kg/day (oral) for 7 days based on three considerations: (i) literature precedent indicating low-to-mid tens of mg/kg/day of MPFF/diosmin are well tolerated and effective in rodent pain/inflammation models; (ii) translational relevance, as clinical MPFF is typically 1000 mg/day, and allometric scaling supports ~50 mg/kg/day as an appropriate rat-equivalent dose for co-therapy with morphine; and (iii) pilot tolerability/signal detection performed in our facility, which confirmed tolerability and a measurable behavioral/biomarker signal at this dose without observable adverse effects. Future dose–response experiments are planned to refine MPFF dosing in the combination setting [33]. On postoperative day 7 following PSNT, an intrathecal catheter and osmotic pump were implanted under 2.0–2.5% isoflurane anesthesia. An ALZET mini-osmotic pump (Model 2001) delivered the drug at the designated doses. A polyethylene catheter (PE-10, 8 cm) was introduced by puncturing the cisternal membrane and advanced caudally to the lumbar enlargement. The rostral end was externalized over the skull, and the incision was closed with sutures. Morphine hydrochloride was dissolved in saline. Daflon was dispersed in water. 30 rats were randomly distributed into 5 groups: Sham, PSNT + Saline, PSNT + Morphine (15 μg/μL/h, i.t.), PSNT + Daflon (50 mg/kg/day, oral), and PSNT + Morphine + Daflon (15 μg/μL/h, i.t. + 50 mg/kg/day, oral) (*n* = 6, each). Morphine was intrathecally administered at a dose of (15 μg/μL/h, i.t.) and Daflon was orally administered for 7 consecutive days.

1. Sham group (uninjured baseline, *n* = 6, saline, i.t.)
2. PNST + Saline (injury control, *n* = 6, saline, i.t.)
3. PSNT + Morphine group (*n* = 6), (15 μg/μL/h, i.t.),
4. PSNT + Daflon group (*n* = 6), (50 mg/kg/day, oral),
5. PSNT + Morphine + Daflon group (*n* = 6), (morphine 15 μg/μL/h, i.t. + oral Daflon 50 mg/kg/day).

### 4.3. Behavioral Measurements

The tail-flick analgesia assay was performed using hot-water immersion at 52 ± 0.5 °C before and after a 5-day morphine infusion. Baseline latency was ~1.95 ± 0.04 s, with a 10 s cutoff to prevent tissue injury. During testing, rats were gently secured in plastic restrainers [34,35].

Tactile allodynia of the left hind paw was assessed with a Dynamic Plantar Aesthesiometer (Ugo Basile, Comerio, Italy). Rats were placed in plastic chambers on a wire-mesh platform and acclimated for 30 min. A blunt 0.5 mm metal probe applied a progressively increasing force (1–50 g) to the plantar surface; three trials were performed at 2 min intervals and averaged. A 50 g cutoff was used [26].

Thermal nociception was measured with a Hargreaves device (Ugo Basile 7371, infrared 80). After 30 min acclimation, rats remained in individual polycarbonate chambers while an infrared stimulus targeted the plantar surface of the left hind paw. Withdrawal latencies were auto-recorded, with trials spaced at 5 min intervals. To avoid burns, a 20 s cutoff was set based on non-operated baseline latencies of 8–10 s [26].

### 4.4. Spinal Cord Sample Preparation

After completing all procedures, rats were euthanized by exsanguination under isoflurane anesthesia (Abbott Laboratories, Queenborough, UK). A laminectomy was performed at the caudal border of T12, and spinal cord segments L5–S3 were rapidly excised, separated into dorsal and ventral portions, and preserved at −80 °C until analysis.

### 4.5. Measurement of Cytokines

Prior to euthanasia, blood was obtained from the tail vein. Samples were centrifuged at 3000× *g* for 15 min to isolate serum, which was stored at −80 °C until analysis. Total serum TNFα, IL-1β, IL-6, and IL-10 were quantified using a colorimetric ELISA kit (Calbiochem-Nocabiochem, Milan, Italy) in line with the manufacturer’s instructions [36]. Marker concentrations were also assessed by radioimmunoassay on a Cobas e411 analyzer, and additional commercial ELISA kits for TNF-α, IL-1β, IL-6, and IL-10 (San Diego, CA, USA; Abcam Co., Cambridge, MA, USA) were utilized. All measurements were performed in triplicate as recommended by the manufacturers. For each independent sample, concentrations were calculated from a standard curve [37,38,39].

### 4.6. H&E Staining for Mononucleate Cell Infiltration in Spinal Cord

The dorsal quadrant of the lumbar spinal cord was carefully isolated and preserved in 4% paraformaldehyde. First, the spinal cords were fixed in 4% paraformaldehyde for 8 h and then cryoprotected in 30% sucrose for an additional 8 h. The spinal cords were paraffinized and sectioned (10 µm). The sections were mounted on glass slides and dried at 37 °C for 1 day before staining. Automated hematoxylin and eosin (H&E) staining was performed using the Leica Autostainer XL (ST5010) (Leica, Wetzlar, Germany). Paraffin-embedded tissue sections were first deparaffinized in xylene (4 times for 5 min each) and then rehydrated through a graded ethanol series (100%, 95%, 90%, 80%, and 70%; 5 min each), followed by a 3 min rinse in 1× PBS. The sections were stained with hematoxylin (either Mayer’s or Harris) for 5 min and rinsed under running tap water twice for 3 min to remove excess dye. Counterstaining was carried out using 0.25% Eosin Y for 1–2 min, followed by two quick rinses in tap water (2 times for 2 min). Slides were then dehydrated in 100% ethanol (3 times for 10 s each), cleared in xylene (3 times for 1 min), and mounted with a xylene-based mounting medium before cover slipping. The stained sections were evaluated under a light microscope, where cell nuclei appeared blue-purple due to hematoxylin, while the cytoplasm, muscle fibers, and red blood cells appeared pink from eosin counterstaining.

### 4.7. Immunohistochemical Analysis for GFAP and Iba-1 in Spinal Cord

The dorsal lumbar cord quadrant was carefully dissected and fixed in 4% PFA (8 h), followed by equilibration in 30% sucrose (8 h). Specimens were paraffin embedded and microtomed into 10 µm sections. Primary staining used GFAP and Iba-1 antibodies (1:100), with a 12 h incubation at 4 °C. Paraffin-embedded tissue slides were first deparaffinized and rehydrated by heating at 60 °C for 5 min and then washed in xylene three times (10 min each). The slides were then sequentially washed for 3 min each in 100% ethanol three times, and then for 1 min each in 95%, 85%, and 75% ethanol. Afterward, slides were rinsed in distilled water for 5 min. For antigen retrieval, slides were immersed in antigen retrieval solution and heated at 120 °C for 2.5 min in a pressure cooker, then cooled to room temperature. Slides were washed three times with distilled water (2 min each). To block endogenous peroxidase activity, tissues were covered with 3% hydrogen peroxide for 15 min, followed by two washes with 1X PBST (2 min each). The primary antibody was diluted using an appropriate antibody diluent as already described, applied evenly to tissue sections, and incubated for 90 min at room temperature. After washing three times with 1X PBST (2 min each), a secondary antibody was applied and incubated for 15 min at room temperature, followed by another PBST washes (three times for 2 min each). Freshly prepared DAB (Leica Biosystems, (Wetzlar, Germany) substrate was then added, and tissues were incubated until suitable staining developed (approximately 5 min), followed by being rinsed in water. Slides were counterstained with Hematoxylin QS for 3 min and rinsed again. Dehydration steps included sequential washes in 75%, 85%, and 95% ethanol for 1 min each, followed by two rinses in 100% ethanol and three washes in xylene (1 min each). Finally, coverslips were mounted using a permanent mounting medium, and slides were dried overnight at room temperature before microscopic analysis. Scanning was carried out with a Leica Aperio AT2 (Leica, Wetzlar, Germany). The resulting images were processed in CaseViewer (Aperio ImageScope Software Version 12.3.2.8013 and CaseViewer Version 2.4) and analyzed quantitatively in ImageJ (Software Version 1.53).

### 4.8. Real-Time Quantitative PCR

At post-implant day 7, rats underwent aortic perfusion with normal saline prior to tissue dissected. The dorsal horn from lumbar segments L4–L6 was isolated. RNA was purified using RNAiso Plus (Takara, Kusatsu, Japan), and cDNA was generated with the PrimeScript RT Reagent Kit containing gDNA Eraser (Takara). qPCR was conducted on a Bio-Rad iCycler with TB Green Premix Ex Taq II (Takara) using 95 °C/5 s and 60 °C/30 s steps. Nrf2 and HO-1 mRNA were analyzed relative to controls, with data presented as mean ± SD.

### 4.9. Statistical Analysis

Mean ± SD is presented throughout. Graphical output and statistics were conducted with GraphPad Prism 9, using two-way ANOVA plus Tukey’s post hoc comparisons and Student’s *t*-tests where applicable.

## 5. Conclusions

This study demonstrates that combining morphine and Daflon offers a clear therapeutic advantage in treating neuropathic pain caused by PSNT injury compared to using either treatment alone. Behavioral tests revealed that the morphine + Daflon significantly improved both mechanical and thermal pain thresholds, suggesting stronger pain-relieving effects. At the molecular level, combination treatment restored antioxidant defenses by increasing the expression of Nrf2 and HO-1, reducing key pro-inflammatory cytokines (TNF-α, IL-1β, and IL-6), and increasing the anti-inflammatory cytokine IL-10. Histological and immunohistochemical analyses further confirmed that morphine + Daflon more effectively reduced mononuclear cell infiltration, astrocyte activation (as indicated by GFAP expression), and microglial activation (as indicated by Iba-1 expression) in the spinal cord compared to either agent alone. These findings suggest that concurrently addressing both oxidative stress and inflammation provides a stronger and more comprehensive neuroprotective strategy. Importantly, combining morphine with Daflon may allow for lower opioid doses, helping to minimize side effects and risks associated with long-term opioid use. Overall, these results support the use of combined morphine and Daflon therapy as a promising and safer approach for managing neuropathic pain while preserving the health and integrity of the spinal cord.

## Data Availability

The original contributions presented in this study are included in the article. Further inquiries can be directed to the corresponding author.
